# Patient Perspectives on the Digitization of Personal Health Information in the Emergency Department: Mixed Methods Study During the COVID-19 Pandemic

**DOI:** 10.2196/28981

**Published:** 2022-01-06

**Authors:** Sophia Ly, Ricky Tsang, Kendall Ho

**Affiliations:** 1 Faculty of Medicine University of British Columbia Vancouver, BC Canada; 2 Department of Emergency Medicine Faculty of Medicine University of British Columbia Vancouver, BC Canada

**Keywords:** emergency medicine, digital health, health informatics, electronic health record, patient portal, patient-physician relationship, COVID-19

## Abstract

**Background:**

Although the digitization of personal health information (PHI) has been shown to improve patient engagement in the primary care setting, patient perspectives on its impact in the emergency department (ED) are unknown.

**Objective:**

The primary objective was to characterize the views of ED users in British Columbia, Canada, on the impacts of PHI digitization on ED care.

**Methods:**

This was a mixed methods study consisting of an online survey followed by key informant interviews with a subset of survey respondents. ED users in British Columbia were asked about their ED experiences and attitudes toward PHI digitization in the ED.

**Results:**

A total of 108 participants submitted survey responses between January and April 2020. Most survey respondents were interested in the use of electronic health records (79/105, 75%) and patient portals (91/107, 85%) in the ED and were amenable to sharing their ED PHI with ED staff (up to 90% in emergencies), family physicians (up to 91%), and family caregivers (up to 75%). In addition, 16 survey respondents provided key informant interviews in August 2020. Interviewees expected PHI digitization in the ED to enhance PHI access by health providers, patient-provider relationships, patient self-advocacy, and postdischarge care management, although some voiced concerns about patient privacy risk and limited access to digital technologies (eg, smart devices, internet connection). Many participants thought the COVID-19 pandemic could provide momentum for the digitization of health care.

**Conclusions:**

Patients overwhelmingly support PHI digitization in the form of electronic health records and patient portals in the ED. The COVID-19 pandemic may represent a critical moment for the development and implementation of these tools.

## Introduction

Patient-centeredness, identified by the Institute of Medicine as one of six pillars of quality care, refers to care that is guided by patient preferences, needs, and values [1]. Although patient-centered approaches in the emergency department (ED) are associated with improved clinical outcomes and patient satisfaction [2-4], they can be challenging when high medical acuity, frequent care transitions, and an unpredictable environment compromise provider-patient communication and collaborative decision-making [5,6].

Personal health information (PHI) digitization is a potential strategy for improving provider-patient communication to support patient-centered care in the ED [6,7]. It encompasses a range of technologies that allow for the collection, analysis, and distribution of digital patient data [8]. These technologies can include electronic health records (EHRs) operated by health care providers as well as EHR-tethered portals for patients to access real-time PHI online.

There has been growing public interest in digital PHI tools. The percentage of Canadian physicians reporting that their patients used digital PHI technologies grew from 20.8% in 2017 to 44.7% by 2019 [9,10], when 74% of Canadian respondents expressed an interest in using patient portals [11]. The COVID-19 pandemic has further encouraged patients and providers to adopt digital health solutions in response to public health guidelines and social distancing requirements [12,13] and has precipitated calls for the widespread integration of digital tools in health care as our systems navigate beyond the COVID-19 crisis [12-14].

Although access to digital PHI has been shown to reduce anxiety, motivate lifestyle changes, and promote patient engagement in the primary care setting [15,16], patient attitudes toward digitization are not well characterized in the emergency setting where patient demographics, priorities, and care journeys may differ [5]. Nonetheless, most EDs in British Columbia (BC), Canada, now use some version of an EHR system that is integrated across the hospital departments within the local health authority and that feeds into CareConnect, a province-wide EHR platform viewable by physicians and other hospital-associated care providers [17]. Laboratory results—but not other EHR components, such as consult notes, imaging reports, and medication orders—are accessible by patients via an online portal [18].

There has been limited work examining the extent to which current digital PHI systems meet the needs of ED users or what opportunities there are to leverage PHI digitization to optimize care delivery in the ED setting. We therefore conducted a mixed methods study to explore the general perspectives of BC ED users on PHI digitization in emergency care.

## Methods

### Participant Recruitment

English-speaking adults aged >19 years who had received care in a BC ED within the last 5 years were invited to complete an online questionnaire via the University of British Columbia Digital Emergency Medicine social media channels, Vancouver Coastal Health Research Institute’s REACH BC directory [19], regional patient networks that shared study details with members, and notices posted in the Vancouver General Hospital ED. Written consent was obtained from all participants.

### Survey

The questionnaire was developed in consultation with 6 patients who have lived ED experiences and a working group of 15 clinicians and researchers brought together through a grant from the Michael Smith Foundation for Health Research in 2019. The questionnaire (Multimedia Appendix 1) included a combination of multiple-choice questions, Likert scales, and free-form text boxes. The eHealth Literacy Scale (eHEALS) [20] was included in the questionnaire to assess participants’ digital health literacy. The questionnaire was administered online via Qualtrics and took approximately 20 minutes to complete. Participants were asked about their demographics, recent experiences in the ED, experiences with digital health technologies, preferences on the use of their digitized ED PHI, and the expected impacts of PHI digitization on the ED experience.

### Key Informant Interviews

Survey participants who indicated that they wished to participate in future activities related to the study were invited by email to provide key informant interviews. Interviews (Multimedia Appendix 2) took place by phone or via the videoconferencing platform Zoom and lasted approximately 30 minutes. Participants were asked about their ED experiences and attitudes toward digital health technologies in the ED. Interviews were audio recorded and transcribed.

### Data Analysis

Survey submissions with more than 20% of items missing were excluded from analysis. Quantitative responses were summarized with descriptive statistics (eg, mean, SD, frequency) and figures were generated using Google Sheets (Google LLC). Statistical tests were not performed as the purpose of our quantitative analysis was to provide a general picture of ED user characteristics and preferences rather than to make comparisons or to identify associations. Qualitative survey and interview responses were analyzed using a conventional content analysis approach wherein codes were defined a posteriori over the course of the analysis [21]. Coding was done independently in NVivo 12 (version 12.6.0; QSR International) by SL and RT, who met regularly to discuss thematic findings. Consensus was achieved for all codes.

## Results

### Participant Demographics

A total of 205 participants responded to the online survey between January and April 2020, of which 108 submissions had <20% of items missing and were included in the final analysis. Of these 108 participants, 16 provided key informant interviews in August 2020. Participant characteristics are summarized in Table 1. Participants were predominantly female (77/108, 71%) and Caucasian (83/108, 77%). Almost all participants reported daily internet (102/107, 95%) and smart device (106/108, 98%) access. Survey and interview participants were comparable in their ED and digital technology experiences, although interview participants reported higher levels of education and income.

Most participants resided within the Lower Mainland of British Columbia (67/108, 62%). In British Columbia, there are 5 geographic health authorities that manage health services in different parts of the province: Vancouver Coastal Health, Fraser Health, Vancouver Island Health, Interior Health, and Northern Health. The distribution of participants who received care from each health authority is also shown in Table 1.

**Table 1:** Participant demographics.

Demographics		Survey (N=108)^a^	Interview (N=16)^a^
Age (years), mean (SD; range)		47.1 (16.8; 19-84)	50.7 (15.9; 21-76)
**Sex, n (%)**			
	Female	77 (71)	12 (75)
	Male	24 (22)	2 (13)
	Other/prefer not to answer	7 (7)	2 (13)
**Ethnicity, n (%)^b^**			
	Caucasian	83 (77)	13 (81)
	East Asian	11 (10)	2 (13)
	Aboriginal	5 (5)	0 (0)
	Latin American/Hispanic	3 (3)	0 (0)
	South Asian	2 (2)	0 (0)
	Other/prefer not to answer	22 (20)	5 (31)
**Education, n (%)**			
	Some high school	1 (1)	0 (0)
	High school diploma	15 (14)	1 (6)
	Trade/technical training	23 (21)	1 (6)
	Bachelor’s degree	34 (31)	8 (50)
	Graduate/professional degree	24 (22)	6 (38)
	Prefer not to answer	11 (10)	0 (0)
**Household income ($), n (%)**			
	<40,000	28 (26)	3 (19)
	40,000-60,000	10 (9)	3 (19)
	60,000-80,000	11 (10)	1 (6)
	80,000-100,000	18 (17)	2 (13)
	>100,000	20 (19)	5 (31)
	Prefer not to answer	21 (19)	2 (13)
**British Columbia health authority in which emergency department care was most recently accessed, n (%)**			
	Vancouver Coastal Health Authority	42 (39)	9 (56)
	Fraser Health Authority	25 (23)	4 (25)
	Vancouver Island Health Authority	19 (18)	1 (6)
	Interior Health Authority	10 (9)	2 (13)
	Northern Health Authority	5 (5)	0 (0)
	Prefer not to answer	7 (6)	0 (0)
**Chronic disease, n (%)**			
	Yes	66 (62)	9 (56)
	No	31 (29)	5 (31)
	I don’t know	10 (9)	2 (13)
Number of emergency department visits in last 5 years, mean (SD; range)		3.4 (2.8; 1-15)	3.0 (2.5; 1-10)
Emergency department visits with altered level of consciousness, n (%)		13 (12)	2 (13)
Emergency department visits with life-threatening medical circumstances, n (%)		32 (30)	5 (31)
**Internet use, n (%)**			
	Daily	102 (95)	16 (100)
	Weekly	3 (3)	0 (0)
	Monthly	0 (0)	0 (0)
	Less than once per month	2 (2)	0 (0)
**Computer, tablet, or smartphone use, n (%)**			
	Daily	106 (98)	16 (100)
	Weekly	1 (1)	0 (0)
	Monthly	0 (0)	0 (0)
	Less than once per month	1 (1)	0 (0)
Past use of digital health technologies, n (%)		87 (81)	14 (88)
eHealth Literacy Scale, mean (SD; range)		33.0 (7.4; 8-40)	32.3 (7.8; 16-40)

^a^Total number of responses may not equal total number of participants as responses were not required for all questions.

^b^Percentages may sum to greater than 100% as participants were able to select multiple responses.

### Survey

**Figure 1:**
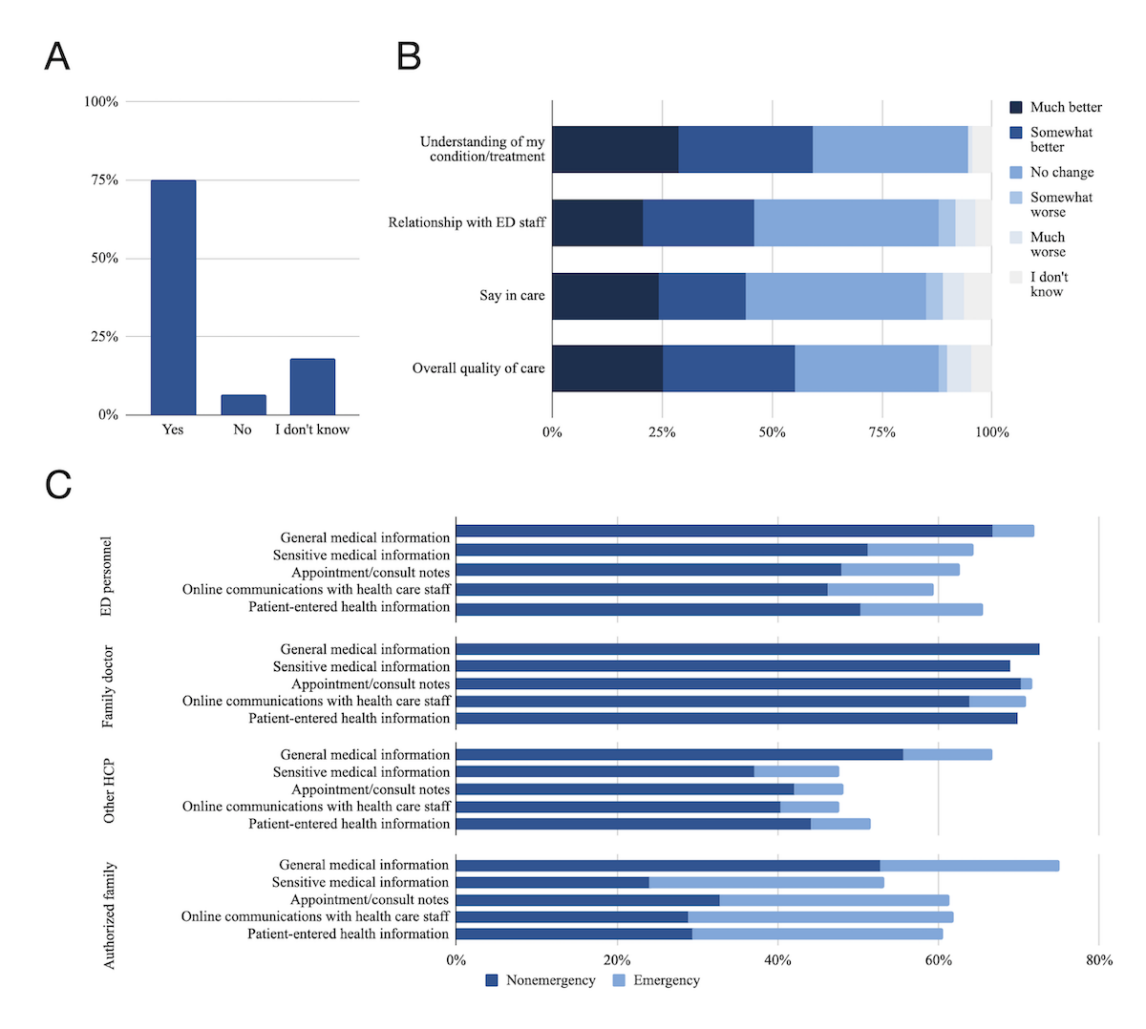
Patient perspectives on ED EHRs. (A) Percentage of respondents who support implementation of EHRs in the ED (N=105). (B) Perceived impacts of EHRs on satisfaction with ED care (N=108). (C) Preferences for ED EHR information disclosure in nonemergency and emergency situations (N=108). "General medical information" refers to test results, diagnoses, and medications. "Sensitive health information" refers to details about sexual health, mental health, and domestic violence. ED: emergency department; EHR: electronic health record; HCP: health care provider.

Figure 1 summarizes participant attitudes toward EHRs in BC EDs. Survey respondents generally supported EHR implementation, with 75% (79/105) in favor, 7% (7/105) against, and 18% undecided (19/105). Respondents expected EHR use to improve their understanding of their medical condition (64/108, 59%), their overall quality of care (59/108, 55%), their relationship with ED staff (50/108, 46%), and their say in care (48/108, 44%). In contrast, 1%-8% (1/108 to 9/108) of respondents expected EHRs to worsen care across these domains. Respondents were generally willing to disclose different components of their EHR to ED staff (68/108, 64% to 90/108, 83% of participants in nonemergencies and 86/108, 80% to 97/108, 90% in emergencies). They were more willing to provide access to their family physicians (83/108, 86% to 98/108, 91% in both nonemergencies and emergencies) and less willing to provide access to designated family/friend caregivers (26/108, 24% to 57/108, 53% in nonemergencies and 57/108, 53% to 81/108, 75% in emergencies). In addition, 73% (79/108) were willing to share deidentified health data with researchers.

When asked about other potential impacts of EHRs in the ED, participants stated that they may provide ED staff with more timely access to relevant PHI (17 respondents) and allow patients to review clinician comments, promoting accountability (2 respondents). In addition, 16 respondents voiced concerns that EHRs increase the risk of unauthorized PHI disclosure, with 5 respondents stating that this was a definite barrier to their support for PHI digitization.

**Figure 2:**
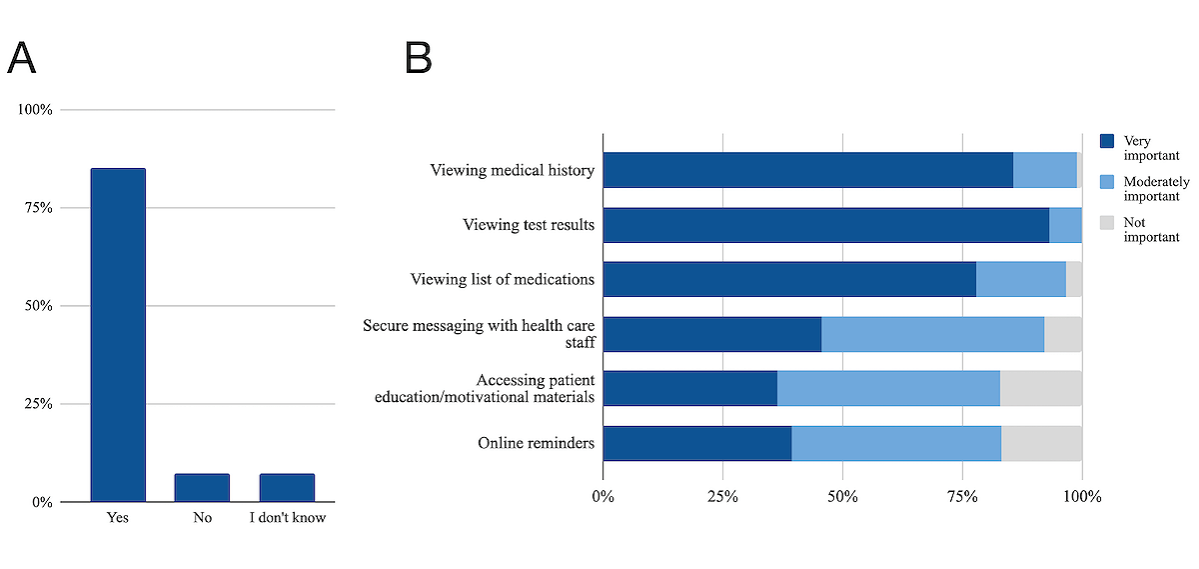
Patient perspectives on ED patient portals. (A) Percentage of respondents interested in using a patient portal to access digitized PHI in their own ED EHR (N=107). (B) Patient-prioritized features for an ED portal (N=91, corresponding to the participants who indicated that they were interested in using a portal to access their ED EHR). ED: emergency department; EHR: electronic health record; PHI: personal health information.

Figure 2 summarizes participants’ views on ED patient portals. Overall, 85% (91/107) of survey respondents were interested in using a portal to access their ED EHR. Of those respondents, 73% (66/91) reported that they would use it in hospital and 100% (91/91) postdischarge. Patient-prioritized features included the ability to view personal medical histories, test results, and medications, which were rated as “very important” by 77% (70/91) to 85% (83/91) of respondents. Some respondents also rated as “very important” the ability to securely message ED staff (41/91, 45%), access patient education or motivational materials (32/91, 35%), and access online reminders (35/91, 38%).

When asked about other potential impacts of patient portals in the ED, participants stated that they would help them to learn about their ED journey (3 respondents), follow discharge instructions (2 respondents), and share information about their visit with community care providers (4 respondents). Participants stated that barriers to portal use include medical incapacity in the ED (6 respondents); limited access to smart devices, internet, or electrical outlets in the ED (5 respondents); limited access to smart devices or the internet in the community (7 respondents); and a challenging user interface (15 respondents).

### Key Informant Interviews

Key informant interviews were conducted to clarify how participants expected PHI digitization to impact ED care. A total of 62 survey participants expressed an interest in being interviewed, of which 16 were ultimately recruited (4 declined, 42 did not respond to follow-up). Of the 16 interviewees, 7 had work experience in health care.

### ED Access to PHI During Emergencies

Multiple factors may limit ED access to past medical information: patients may be unable to share PHI due to medical incapacity or emotional stress (7 interviewees), collateral may be incomplete (1 interviewee), and patients may not be trusted to provide accurate information concerning controversial diagnoses (eg, Ehlers-Danlos syndrome) without documentation (2 interviewees).

Several participants expected PHI digitization to enhance history-taking by facilitating ED access to data stored in an EHR integrated between EDs and other health services (9 interviewees). One interviewee expressed surprise upon learning that BC EDs did not already have access to her family physician’s electronic records:

When I realized that the hospital didn’t have my health history digitally when they did that intake a couple years ago, I was like, oh my gosh. People think that their health is saved more digitally at their doctor’s office and in the hospital than it actually is.

Improved ED access to patient medical histories was expected to increase the efficiency of face-to-face patient-physician interactions (13 interviewees) and promote confidence in the quality of care received (2 interviewees). Multiple participants, however, expressed concern that digitization could facilitate unauthorized access to PHI by corporations or health professionals not involved in their care (6 interviewees).

### Relationship Between Patients and ED Staff

Interviewees suggested that relationships between patients and ED staff can be undermined when physical discomfort (1 interviewee), anxiety (2 interviewees), or feelings of being neglected during long wait times (2 interviewees) contribute to high tensions during in-person interactions. There were also concerns about poor accountability from ED staff in cases of medical error or professional misconduct (3 interviewees).

Participants generally expected relationships with ED staff to improve with PHI digitization (10 interviewees). By updating patients on their medical status in real time, ED portals may alleviate anxiety ahead of face-to-face interactions with care providers (2 interviewees) and offer a glimpse of behind-the-scenes care processes, providing reassurance that patients are not forgotten during their visit (2 interviewees). As one interviewee stated, “If I know the reason why I’m waiting in the emergency room is because they’re just waiting for results and diagnostics... I know what I’m waiting for and don’t feel like I’ve been deprioritized.”

Two interviewees described how patient-ED relationships may worsen with PHI digitization. One stated that electronic access to historic medical records may facilitate the disclosure of stigmatizing information (eg, psychiatric conditions), biasing providers against patients. The other interviewee, a former ED nurse, indicated that digital technologies may detract from the human aspect of care:

When you improve efficiency, you kind of lose the art of…from my perspective, nursing. Where you take the time to put a warm blanket on, to hold somebody’s hand, to help them with their dentures, whatever is required.

Participants also suggested that patient portals could be a tool for improving accountability from ED staff. Portals may allow patients to identify errors or discriminatory remarks in their chart (3 interviewees). One interviewee suggested that the opportunity for patients and providers to participate in mutual surveillance may deconstruct the power imbalance inherent in clinical relationships.

### Self-advocacy in the ED

Several interviewees described how patient self-advocacy in the ED can be compromised by insufficient opportunity to process information from health professionals, with one participant stating:

A lot of what happens in healthcare is a one-way conversation. It’s almost as an afterthought at the end of a whole bunch of information spewing towards you – do you have any questions? And you don’t have enough time to really think about it and digest what you just heard to formulate a question quickly, especially if you’re in the emergency department in pain.

Concern about interrupting the ED workflow was also identified as a barrier to self-advocacy. One interviewee stated that she did not receive analgesia until the end of her visit as she did not know the appropriate way to voice her concern and “just didn’t feel like bothering anyone.”

Patient portals in the ED may allow patients to learn about their medical status ahead of in-person encounters, facilitating more informed decision-making (6 interviewees). Portals may also provide a nonintrusive process for bringing up care concerns, increasing the likelihood that they will be voiced (2 interviewees). Barriers to their use in the ED include medical incapacity (8 interviewees) and limited access to smart devices (1 interviewee), which may be minimized through patient-accessible smart devices in the ED or user controls authorizing portal access by designated family members during emergencies.

### Self-management After the ED

Participants indicated that ED patients have limited access to visit details for postdischarge self-management. Medical incapacity and emotional stress can prevent patients from recalling visit details presented verbally by care providers (4 interviewees) and incidental findings are not consistently shared with patients (3 interviewees).

ED portals were suggested to enhance patients’ understanding of their medical condition at discharge (14 interviewees), increase compliance with discharge instructions (5 interviewees), and facilitate online self-education (5 interviewees). One respondent remarked that visitor restrictions due to the COVID-19 pandemic made it more important for patients cognitively impaired by pain or illness to have a digital record of their visit postdischarge. Digital access to ED test results may also allow for follow-up of incidental findings. Two interviewees stated that they were diagnosed with medical conditions that could have been identified earlier had they been informed of abnormal results obtained in the ED.

Digital ED PHI access was expected to enhance information-sharing with family caregivers, allowing them to better support patients in decision-making and day-to-day care implementation (eg, transport to appointments; 3 interviewees). Digitization was also expected to improve information-sharing with allied health professionals, giving patients more autonomy in where they seek postdischarge care (6 interviewees).

Potential barriers to effective portal use postdischarge may include limited access to smart devices or the internet, particularly for rural-dwelling or low-income patients (7 interviewees), as well as difficulties using the portal interface or interpreting medical information (14 interviewees).

### Effects of the COVID-19 Pandemic on Patient Attitudes Toward Digital PHI Technologies

In total, 6 interviewees stated that the COVID-19 pandemic has highlighted the importance of digital health technologies in modern health care delivery. In addition, 4 further expressed that the COVID-19 pandemic has provided government and health care organizations with the impetus to enact these technologies, with 1 participant describing how First Nations reservations in the BC Interior have recently established high-speed internet infrastructure to facilitate telehealth consultations.

## Discussion

### Principal Findings

Our findings suggest that the majority of participants are supportive of ED PHI digitization in the form of EHR and patient portal implementation. The anticipated benefits of PHI digitization on the patient emergency care experience can be grouped into four domains: (1) overcoming challenges of the ED environment by relieving anxiety and fostering relationships with staff, (2) facilitating access to information by ED staff and patients, (3) promoting self-advocacy by enhancing patient decision-making capacity and health care provider accountability, and (4) easing care transitions by facilitating medical self-management, self-education, and care planning with community providers. Users were interested in portal features consistent with these aims.

Although this is the first study to our knowledge that examines the perspectives of ED users on PHI digitization, these findings are consistent with primary care studies suggesting that portals can alleviate anxiety [22], increase patient activation [15,22], and facilitate collaborative relationships with clinicians [23,24]. Our results differ from those of previous studies by identifying barriers to portal use that are specific to the ED context, such as high medical acuity or difficulties with in-hospital internet and smart device access. In addition, whereas previous work in the primary care context found that patient engagement in portals is contingent upon a pre-existing foundation of trust between patients and their providers [25], our results suggest that patient portals may work inversely in the emergency setting to foster trust in new providers.

Although the participants in our study were generally enthusiastic about PHI digitization and patient portals in the ED, positive perception may not translate to actual portal uptake. A recent study from the University of Iowa reported that only 8.9% of ED users used a portal to view their test results, possibly due to a lack of multilingual settings, internet and smart device access, or patient education on portal use [26]. It is therefore incumbent upon institutions to consult patients as stakeholders in the development of digital PHI tools and care providers to meaningfully engage patients in their use.

The minority of participants who opposed ED PHI digitization expressed concerns over information privacy and security. The potential for PHI compromise through third-party breaches or unauthorized release to employers or insurance companies is a common theme among studies exploring barriers to portal use [27]. Mitigation strategies include data minimization, encryption policies, proxy accounts providing family caregivers with access to preauthorized content, and audit trails allowing patients to view users who have accessed their EHR [28]. To safeguard patient confidence in digital PHI systems, the Canadian Medical Protective Association also recommends patient counselling on safe data practices and provider transparency regarding who has PHI access [29].

A major limitation of this study is that self-selection bias may have led to an overrepresentation of positive attitudes toward PHI digitization. Although our open recruiting strategy makes it challenging to determine the extent to which our survey cohort is representative of the general population of BC ED users, among our interview participants, 7 of 16 reported work experience in health care. There is evidence that health care workers self-report high levels of digital literacy and share homogenous, generally positive viewpoints toward PHI digitization [30]. Similarly, the perspectives of vulnerable and marginalized populations (eg, low socioeconomic status) were underrepresented in this study. Several interviewees stated that these populations may have unique perspectives on digitization, a suggestion supported by previous findings that lower engagement in eHealth activities is associated with lower socioeconomic status, ethnic minority status, and rural residency [31]. Future work should seek to capture the perspectives of a broader range of ED users to inform the creation of equitable digital PHI tools.

As of September 2021, COVID-19 continues to impact the global community. In British Columbia, a resurgence of cases emerged in July 2021 but began to stabilize as of late August 2021, with daily reported cases exceeding 600 in early September 2021 [32]. As we completed data collection in August 2020, we were unable to capture the ongoing effects of the COVID-19 pandemic on evolving patient attitudes in British Columbia. However, participant observations that the COVID-19 pandemic has spurred the health care system to implement overdue digital reforms allow us to hypothesize that support for PHI digitization is likely to remain robust as the global pandemic evolves.

### Conclusion

Our findings suggest that BC ED users welcome PHI digitization and expect it to enhance their ED experience by increasing patient comfort, facilitating communication with ED health professionals, and improving post-ED care. The COVID-19 pandemic provides a window of opportunity for introducing digital PHI technologies to improve ED care as part of the larger digital revolution currently affecting health care internationally.

## Appendix

Multimedia Appendix 1 Survey questionnaire.

Multimedia Appendix 2 Interview questions.

## Abbreviations

BC: British Columbia
ED: emergency department
eHEALS: eHealth Literacy Scale
EHR: electronic health record
PHI: personal health information
