# Supplementary material for: Patient Perspectives on the Digitization of Personal Health Information in the Emergency Department: Mixed Methods Study During the COVID-19 Pandemic
Source: JMIR Med Inform. 2022 Jan 6;10(1):e28981. doi: 10.2196/28981 (PMC8734606; doi:10.2196/28981)
Supplement: Multimedia Appendix 1 [file medinform_v10i1e28981_app1.docx]

**Section 1: Your Experience with the Emergency Department (ED)**

Have you visited an ED in B.C. within the last 5 years?

- Yes
- No

In the last 5 years, how many times have you visited the ED as a patient?

When was your most recent visit to an ED in B.C. as a patient?

- Less than 3 months ago
- 3-6 months ago
- 6 months - 1 year ago
- 1-3 years ago
- 3-5 years ago

Which ED in B.C. did you visit most recently as a patient?

During your last ED visit, did you enter the hospital under life-threatening, emergency circumstances?

- Yes
- No
- I don't know

During your last ED visit, were you unconscious or confused when you entered the hospital?

- Yes
- No
- I don’t know

Please give us a brief summary (max. 3 sentences) of what happened during your last ED visit. For example: What brought you in? What was the diagnosis? What treatment(s) did you receive?

Reflecting on your most recent experience in the ED, indicate whether you agree with the following statements. If a statement does not apply to you or you do not know, please select "N/A".

- I was satisfied with my understanding of my condition/treatment.
- I was able to get in touch with healthcare staff when I had questions.
- I was able to discuss anxieties about my condition/treatment with healthcare staff.
- I was comfortable sharing sensitive information with healthcare staff.
- The information I received from different healthcare professionals was consistent.
- I was able to communicate with friends/family during the visit.
- I was able to accurately communicate medical information from my visit with friends/family and non-ED care providers after discharge.
- Overall, I had enough say in my care.
- Overall, I was satisfied with my relationship with the healthcare staff.
- Overall, I was satisfied with the quality of my care.

*Options: Strongly disagree, Disagree, Neutral, Agree, Strongly agree, N/A.*

If you strongly disagreed with any of the statements from the previous question, please explain in the box below.

**Section 2: Your Experience with Digital Health Technologies**

How often do you use each of the following digital technologies? Select the option that best applies to you.

- Internet
- Computer (desktop or laptop)
- Tablet (e.g. iPad)
- Smart phone (e.g. iPhone, Android)

*Options: Never, Less than once per month, Monthly, Weekly, Daily.*

Have you used digital tools (e.g. mobile applications or websites) for your health and wellness before?

- Yes
- No
- I don't know

What did you use these digital tools for?

- Managing diet and/or exercise
- Managing medications and/or appointments
- Tracking symptoms and/or health information
- Reviewing lab results
- Attending medical appointments over video conference
- Other:

When did you first use digital tools for your health and wellness?

- Less than 1 month ago
- 6 months - 1 month ago
- 1 year - 6 months ago
- More than 1 year ago

Are you still using at least one of these digital tools?

- Yes
- No

Indicate whether you agree or disagree with the following statements:

- I know how to find helpful health resources on the Internet.
- I know how to use the Internet to answer my health questions.
- I know what health resources are available on the Internet.
- I know where to find helpful health resources on the Internet.
- I know how to use the health information I found on the Internet to help me.
- I have the skills I need to evaluate the health resources I find on the Internet.
- I can tell high quality from low quality health resources on the Internet.
- I feel confident in using information from the Internet to make health decisions.

*Options: Strongly disagree, Disagree, Neutral, Agree, Strongly agree.*

**Section 3: Digital Health Technologies in the Emergency Department (ED)**

In British Columbia, patient health records in the ED are largely paper-based. There is interest in transitioning from traditional paper-based records to electronic health records, or EHRs. EHRs would allow data to be analyzed for trends or shared online with patients, friend/family caregivers, and healthcare providers through mobile phone applications or websites called patient portals. Some portals have additional functions, allowing patients to message their doctors directly, schedule appointments, or learn more about their medical conditions. Reflecting on your most recent ED visit, how do you think a patient-accessible EHR would have affected each of the following? Please select N/A if you are unsure.

- Your ability to get in touch with healthcare staff when you had questions
- Your ability to discuss anxieties about your condition/treatment with healthcare staff
- Your comfort sharing sensitive information with healthcare staff
- Communication between healthcare staff
- Your ability to communicate with friends/family during the visit
- Your ability to accurately share medical information from your visit with friend/family caregivers or non-ED care providers after discharge
- The amount of say you had in your care
- Overall relationship with healthcare staff
- Overall quality of care

*Options: Much worse, Somewhat worse, No change, Somewhat better, Much better, N/A.*

Are there any other ways that an EHR may have impacted your ED experience?

- Yes - briefly explain:
- No

Who should have access to the different sections of your Emergency Department electronic health record (ED EHR) under non-emergency circumstances? Check all that apply.

- General medical information (e.g. test results, diagnoses, medications, procedures)
- Sensitive medical information (e.g. sexual health, mental health, domestic violence)
- Appointment and consultation notes
- Online communications with healthcare staff
- Patient-entered health information

*Options: You, ED personnel, Family doctor, Other healthcare professionals, Authorized family/friend caregiver.*

Who should have access to the different sections of your ED EHR during a medical emergency if you are unconscious or confused? Check all that apply.

- General medical information (e.g. test results, diagnoses, medications, procedures)
- Sensitive medical information (e.g. sexual health, mental health, domestic violence)
- Appointment and consultation notes
- Online communications with healthcare staff
- Patient-entered health information

*Options: You, ED personnel, Family doctor, Other healthcare professionals, Authorized family/friend caregiver.*

For what reasons would you be willing to share components of your EHR with healthcare professionals? Choose all that apply.

- To give emergency personnel timely access to important medical information
- To facilitate communication between the ED and my family doctor
- Other:

For what reasons would you be willing to share components of your EHR with your friend/family caregivers? Choose all that apply.

- For help coordinating my day-to-day care
- For help coordinating my care during an emergency
- For help using my EHR or understanding the information in it
- To communicate with my caregivers when I am in the ED
- Other:

For what reasons would you be unwilling to share components of your EHR? Choose all that apply.

- Others do not need to know
- I'm concerned about the privacy of my information
- I'm concerned about prejudice or discrimination
- Other:

Would you be willing to share information from your ED EHR, with names and other identifying details removed, with health researchers?

- Yes
- No
- I don't know

Wearable devices (“wearables”) are non-invasive electronic sensors that collect health data (e.g. blood pressure, heart rate, body temperature) continuously and in real time. Common examples include fitness trackers (e.g. smartwatch, Fitbit) and continuous glucose monitors. Information from wearables can be analyzed for trends. Have you ever used a wearable device in your day-to-day life?

- Yes
- No
- I don’t know

Do you think it would be helpful for medical professionals in the ED to have access to some of your day-to-day wearable data?

- Yes
- No
- I don't know

Has your health status ever been monitored by medical professionals in the ED using a wearable device?

- Yes
- No
- I don’t know

Would it be helpful for you to have access to the wearable data collected during your ED visit?

- Yes
- No
- I don't know

How would you use the data? Select all that apply.

- To understand my condition in the ED
- To help my family doctor or friend/family caregivers to understand my condition in the ED
- To manage my health after I return home
- Other:

Do you think that wearable data should be routinely collected and used in the ED?

- Yes
- No
- I don’t know

Please use this text box if you would like to provide a brief explanation.

EHRs are centralized, provincially-managed electronic records. As a result, there are laws in place to protect health information in EHRs from inappropriate access, use, and disclosure. Are you familiar with your rights under B.C.'s current health privacy laws?

- Extremely familiar
- Very familiar
- Moderately familiar
- Slightly familiar
- Not familiar at all

Are you confident that B.C.'s current health privacy laws will be able to keep the information in your ED EHR safe?

- Yes
- No
- I don't know

What effect do you expect EHRs to have on the privacy of your ED health information?

- Better privacy
- No change
- Worse privacy
- I don't know

Drag and drop the following privacy features in order of what you consider most important to least important for your ED EHR.

- Controlling who can access which components of your EHR
- Limiting access to some components of your EHR under non-emergency circumstances
- Having a list of who has accessed what information
- Other:

Do you think that the advantages of EHR use in the Emergency Department (ED) outweigh its disadvantages?

- Yes
- No
- I don’t know

Do you support the implementation of EHRs in EDs in British Columbia?

- Yes
- No
- I don't know

Please use this text box if you would like to provide a brief explanation.

Would you be interested in using a patient portal to access your ED EHR and/or to manage your ED care?

- Yes
- No
- I don't know

What would you like to do with your ED patient portal? Indicate whether the following portal functions are very important, moderately important, or not important to you.

- Viewing medical history
- Viewing test results Viewing list of medications
- Viewing immunization records
- Printing out EHR
- Sharing health information with family/friends
- Secure messaging with healthcare staff
- Online appointment booking
- Online prescription refill requests
- Tracking health data
- Accessing patient education/motivational materials
- Online calendars and reminders

Are there other features that you would be interested in using?

- Yes - briefly explain:
- No

When would you use the portal? Choose all that apply.

- In hospital
- After I go home from hospital

What might prevent you from using the portal effectively?

**Section 4: About You**

What is your age?

What city or town do you live in?

What gender do you identify with?

- Male
- Female
- Other:
- Prefer not to answer

What is your ethnicity? You may select more than one option.

- Aboriginal (First Nations, Metis, Inuit) Arab
- Black (African or Caribbean)
- East Asian (Chinese, Japanese, Korean) Pacific Islander
- Latin American/Hispanic
- South Asian (Indian, Pakistani, Sri Lankan)
- South East Asian (Vietnamese, Cambodian, Laotian) West Asian (Iranian, Afghan)
- Caucasian
- Other:
- Prefer not to answer

What is the highest level of education that you have completed?

- Some high school, no diploma
- High school diploma
- Trade/technical/vocational training
- Bachelor's degree
- Graduate/professional degree
- Prefer not to answer

What is your annual household income?

- Less than $40,000
- $40,000 - $60,000
- $60,000 - $80,000
- $80,000 - $100,000
- More than $100,000 Prefer not to answer

What is your preferred language?

- English
- French
- Spanish
- Punjabi/Hindi
- Chinese (Cantonese/Mandarin)
- Other:

A chronic disease lasts more than three months and gets progressively worse without treatment. Have you been diagnosed with at least one chronic disease?

- Yes
- No
- I don't know

Are you interested in providing further input on the development of patient-accessible EHRs for ED patients in British Columbia?

- Yes
- No

Please indicate your interest in participating in any of the following:

- Future surveys
- Future focus groups
- Future interviews

Please provide your email address or phone number so that we can contact you in the future. Your contact information will be separated from the rest of your survey response.

How did you hear about our survey?

- Word of mouth
- Social media (e.g. Facebook, Twitter)
- Craigslist/Kijiji
- Email
- Flyers
