# Supplementary material for: Patient Perspectives on the Digitization of Personal Health Information in the Emergency Department: Mixed Methods Study During the COVID-19 Pandemic
Source: JMIR Med Inform. 2022 Jan 6;10(1):e28981. doi: 10.2196/28981 (PMC8734606; doi:10.2196/28981)
Supplement: Multimedia Appendix 2 [file medinform_v10i1e28981_app2.docx]

1. **Participant background.**

*Key question:*

- Describe your general health and past experiences as a patient in the emergency department.

*Additional probing questions:*

- How do you feel about your ability to share medical information with emergency staff during the visit(s)?
- How do you feel about the ability of emergency staff to share medical information with you during the visit(s)?

1. **Effect of PHI digitization on information transfer in the ED.** As a reminder, patient portals are web applications connected to electronic health records. They allow patients to access their health information online and in real-time. Some portals have additional functions that allow patients to message their doctors directly, schedule appointments, or learn more about their medical conditions.

*Key question:*

- How do you expect a patient portal to impact information-sharing between you and emergency staff?

*Additional probing questions:*

- How will it affect your ability to share medical information with emergency staff?
- How will it affect the ability of emergency staff to share medical information with you?
- What kind of information would you like to receive from emergency staff through a portal?
- How will a portal affect your face-to-face interactions with emergency staff?

1. **Effect of PHI digitization on engagement in care in the ED.** Survey respondents generally expected that access to a patient portal would help them to understand their medical condition. However, they did not expect that this would give them a greater say in their care.

*Key question:*

- How do you expect patient portal with your emergency department data to impact your understanding of your medical condition and your say in care?

*Additional probing questions:*

- Could you comment on why having a better understanding of your medical condition and treatment may or may not translate to having a greater say in your care in the emergency department?

1. **Effect of PHI digitization on self-management in the community.** More of our survey participants said they would use an emergency department patient portal after discharge rather than in hospital.

*Key question:*

- How would you use a patient portal with your emergency department data after you’ve been discharged home?

*Additional probing questions:*

- What effect, if any, would a patient portal with your emergency department data have on how you manage your chronic medical conditions in the community?

1. **Barriers to use of digitized PHI**. While most of the survey participants are comfortable using digital technologies like computers and smartphones, many of them were still concerned about their ability to interact with the portal interface or to understand the information in it.

*Key question:*

- What supports would you need to meaningfully use the information in your patient portal?

*Additional probing questions:*

- What educational supports would help you use a patient portal?
- What technological supports would help you to use a patient portal?

1. **Use of wearable data in the ED.** Wearables are non-invasive electronic sensors that collect health data like blood pressure or heart rate continuously and in real time. Common wearables include fitness trackers like smartwatches and Fitbits.

*Key question:*

- How do you feel about the use of wearable data in emergency care?

1. **Effect of COVID-19 on patient attitudes towards technology use.**

*Key question:*

- The next question will ask about your experiences with COVID-19. This is a stressful topic for a lot of people, and we can skip this question if you don’t feel comfortable talking it. How has the COVID-19 situation affected your perspective on the use of digital technologies in healthcare?

1. **Closing.** Is there anything else you’d like to share?
